# Trust Deficit in Surgical Systems in an Urban Slum in India Under Universal Health Coverage: A Mixed Method Study

**DOI:** 10.3389/ijph.2022.1604924

**Published:** 2022-07-14

**Authors:** Kranti Vora, Shahin Saiyed, Dileep Mavalankar, Lyndsay S. Baines, Rahul M. Jindal

**Affiliations:** ^1^ Indian Institute of Public Health, Gandhinagar, India; ^2^ Director of Indian Institute of Public Health, Gandhinagar, India; ^3^ Head of School (Health and Social Care), Anglia Ruskin University, London, United Kingdom; ^4^ Professor of Surgery and Global Health, Uniformed Services University, Bethesda, MD, United States; ^5^ Adjunct Indian Institute of Public Health, Gandhinagar, India

**Keywords:** universal health coverage, unmet surgical need, low and middle-income countries, gender discrimination, trust and perception of quality, India

## Abstract

**Objectives:** We carried out a mixed method study to understand why patients did not avail of surgical care in an urban slum in India.

**Methods:** In our earlier study, we found that out of 10,330 people, 3.46% needed surgery; 42% did not avail of surgery (unmet needs). We conducted a follow-up study to understand reasons for not availing surgery, 141 in met needs, 91 in unmet needs. We administered 2 instruments, 16 in-depth interviews and 1 focused group discussion.

**Results:** Responses from the 2 groups for “the Socio-culturally Competent Trust in Physician Scale for a Developing Country Setting” scale did not have significant difference except for, prescription of medicines, patients with unmet needs were less likely to agree (*p* = 0.076). Results between 2 groups regarding “Patient perceptions of quality” did not show significant difference except for doctors answering questions where a higher proportion of unmet need group agreed (*p* = 0.064). Similar observations were made in the in depth interviews and focus group.

**Conclusion:** There is a need for understanding trust issues with health service delivery related to surgical care for marginalized populations.

## Introduction

Surgery has become an integral part of public health with an estimated 234 million operations performed yearly. Surgical intervention is required to promote, prevent and restore health, in every age group [1]. The issue of surgical unmet needs to be addressed at the macro and micro levels. At the macro level, governance and national policy are important [2, 3] while fear of anaesthesia, lack of local social support, difficulty navigating the healthcare system, expensive travel, and lack of privacy have been implicated for low uptake of free essential surgical procedures [4]. Other potential factors at the micro-level are poor health literacy, trust deficit, lack of informed consent which come into play during their decision not to proceed for surgery [5].

The current study is an in-depth exploration of our initial study [6] in which we studied 10,330 populations from the urban slums of Ahmedabad city in the state of Gujarat, India in 2019–20 [6]. Out of 3.46% of surgical need, 42% did not avail surgical care needed for a variety of reasons. Financial reasons (34.5%) and lack of trust (35.3%) were major reasons for not availing essential surgical care despite obtaining consultation from qualified surgeons under India’s universal health coverage (UHC) (https://www.india.gov.in/spotlight/ayushman-bharat-national-health-protection-mission).

We carried out an instrument-based quantitative survey supplemented by a qualitative study to understand the reasons for patients not availing of surgical procedures despite widespread awareness of UHC in India [7]. We focused on the doctor-patient relationship and broader social considerations, which may impact their decision-making. Focus on the doctor-patient relationship has primarily manifested in the form of trust [8] control [9], perceptions of quality [10]. The findings of the study could be used to strengthen surgical systems in India and other LMICs.

## Methods

We contacted 274 people with surgical needs from our original study [6] to participate in exploration of their experience using standardized instruments validated in the Indian population—232 participants agreed out of which, 141 were from met needs, and 91 from the unmet needs group.

Study Instruments: We administered 2 instruments “the Socio-culturally Competent Trust in Physician Scale for a Developing Country Setting” [11, 12] and “Patient perceptions of quality” [13]. The Socio-culturally Competent Trust in Physician Scale for a Developing Country Setting [9] is an excellent tool to study the micro aspects of surgeon-patient interaction and issues which impact upon the low uptake of surgical intervention. The second instrument “Patient perceptions of quality” was administered to understand patients’ viewpoints if there were specific reasons why ∼50% of patients refused surgical procedures despite availing consultation under UHC.

Qualitative study: A subgroup of participants was individually interviewed (*n* = 16) and one focus group discussion (*n* = 5) was done. The subgroup was a mix of met (*n* = 7) and unmet needs (*n* = 14). Supplementary Appendix S2 gives the set of questions asked for the qualitative part of the study.

Data collection: The tools were translated into Gujarati by certified translators (Supplementary Appendix S1) and data was collected on tablets. A web-based application (ODK) was developed for data collection.

Study Analysis: Quantitative analysis was done using statistical software SPSS version 23. Descriptive analysis was used to assess prevalence of surgical conditions and the unmet needs. As the data was homogenous, regression analysis could not be done. Also the difference between both the groups was not major, so we could not do multivariate analysis. P values were obtained to see differences between met need and unmet need patients. Qualitative data was analysed using ‘Framework Method’ as described by Gale et al. [14]. We used the same domains as in the quantitative tools. We used this particular methodology as it is flexible and can generate themes which could be analysed by multi-disciplinary research teams that comprise of healthcare professionals, psychologists, sociologists, economists, and service users, as in our study.

## Results

Demographic information are given in Table 1. 70% had less than 10 years of education. 45% were housewives while 28% were daily wagers. As shown in Table 2, the met needs group was younger with higher proportion of males. The unmet needs group had a higher proportion of illiterate persons and housewives. Except for gender (*p* = 0.007), there was no statistically significant difference between both groups.

**TABLE 1:** Descriptive statistics of baseline characteristics and demographics (Ahmedabad, India. 2022).

N = 232	Frequency	Percent
Age of participants		
14–17 years	9	3.9
18–24 years	15	6.5
25–34 years	36	15.5
35–44 years	48	20.7
45–54 years	48	20.7
55–64 years	44	19.0
More than 64 years	32	13.8
Gender		
Male	102	44.0
Female	130	56.0
Education		
Illiterate	82	35.3
Primary school	81	34.9
Higher secondary	51	22.0
12th Pass	11	4.7
More than 12 std	7	3.0
Occupation		
Own Business	1	0.4
Job	25	10.8
Daily wager	66	28.4
Pensioner/Old age benefit	6	2.6
Unemployed	19	8.2
Student	10	4.3
Housewife	105	45.3
Met or unmet need		
Met need	141	60.8
Unmet need	91	39.2

**TABLE 2:** Comparison between met and unmet needs (Ahmedabad, India. 2022).

	Met need		Unmet need		P-value
	Frequency	Percent	Frequency	Percent	
Age of participant					
14–17 years	7	5.0	2	2.2	0.154
18–24 years	11	7.8	4	4.4	
25–34 years	25	17.7	11	12.1	
35–44 years	32	22.7	16	17.6	
45–54 years	23	16.3	25	27.5	
55–64 years	26	18.4	18	19.8	
More than 64 years	17	12.1	15	16.5	
Gender					
Male	72	51.1	30	33.0	0.007
Female	69	48.9	61	67.0	
Education					
Illiterate	44	31.2	38	41.8	0.201
Primary school	57	40.4	24	26.4	
Higher secondary	28	19.9	23	25.3	
12th Pass	7	5.0	4	4.4	
More than 12 std	5	3.5	2	2.2	
Occupation					
Own business	1	0.7	0	0.0	0.215
Job	17	12.1	8	8.8	
Labour work	44	31.2	22	24.2	
Pension/old age benefit	4	2.8	2	2.2	
Without job	13	9.2	6	6.6	
Student	8	5.7	2	2.2	
House wife	54	38.3	51	56.0	

Table 3 compares the two groups for the “the Socio-culturally Competent Trust in Physician Scale for a Developing Country Setting” scale. Both groups were similar and did not have statistically significant differences except for, prescription of medicines, where unmet needs was less likely to agree (*p* = 0.076).

**TABLE 3:** Comparison between met needs and unmet needs for the “socio-culturally competent trust in physician scale for a developing country Setting” (Ahmedabad, India. 2022).

	Met need (N = 141)	Unmet need (N = 91)	P value		Met need (N = 141)	Unmet need (N = 91)	P value
	Percent	Percent			Percent	Percent	
The doctor does appropriate blood tests and other tests to diagnose my disease				The doctor prescribed more expensive medicines for serious illnesses			
Disagree	1.4	4.4	0.377	Disagree	13.5	13.2	0.964
Neutral	2.1	2.2		Neutral	7.8	8.8	
Agree	96.5	93.4		Agree	78.7	78.0	
The doctor gives appropriate medications for my disease				The doctor prescribes appropriate number of medicines based on the nature of the illness			
Neutral	1.4	4.4	0.163	Neutral	1.4	5.5	0.076
Agree	98.6	95.6		Agree	98.6	94.5	
The doctor’s treatment relieves the illness quickly				The illness gets relieved with just one visit, there is no need for repeat visits			
Disagree	1.4	2.2	0.553	Disagree	6.4	7.7	0.818
Neutral	6.4	9.9		Neutral	8.5	6.6	
Agree	92.2	87.9		Agree	85.1	85.7	
There are no side effects to the medicines prescribed by the doctor				Friends, relatives and neighbours speak well about the treatment provided by the doctor			
Disagree	1.4	0.0	0.453	Disagree	5.0	1.1	0.287
Neutral	2.1	3.3		Neutral	9.9	9.9	
Agree	96.5	96.7		Agree	85.1	89.0	
Friends, relatives and neighbours recommend me to go to the doctor				I get the confidence that all my illness will get alright when I go to the doctor			
Disagree	2.8	1.1	0.536	Disagree	5.0	2.2	0.385
Neutral	6.4	4.4		Neutral	4.3	2.2	
Agree	90.8	94.5		Agree	90.8	95.6	
There is a big crowd in the clinic of the doctor				If I go to the doctor, I will surely get good treatment for my illness			
Disagree	2.8	2.2	0.69	Disagree	2.1	0.0	0.09
Neutral	0.7	0.0		Neutral	2.1	6.6	
Agree	96.5	97.8		Agree	95.7	93.4	
The doctor gives me good treatment irrespective of whether I have money to pay				The main intention of the doctor is to treat my illness and not anything else			
Disagree	41.8	33.0	0.299	Disagree	11.3	8.8	0.809
Neutral	18.4	25.3		Neutral	9.9	11.0	
Agree	39.7	41.8		Agree	78.7	80.2	
Irrespective of what time of the day it is, whenever I go, I can get good treatment from the doctor				I respect the doctor a lot			
Disagree	6.4	3.3	0.321	Disagree	0.7	1.1	0.951
Neutral	5.0	8.8		Neutral	2.1	2.2	
Agree	88.7	87.9		Agree	97.2	96.7	
I think the doctor is a very learned person				I admire the doctor			
Disagree	0.7	0.0	0.522	Disagree	0.7	1.1	0.587
Neutral	0.7	0.0		Neutral	0.7	2.2	
Agree	98.6	100.0		Agree	98.6	96.7	

Table 4 shows the comparison between two groups for “Patient perceptions of quality” instrument. There was no statistically significant difference between met and unmet needs groups except for doctors answering questions where a higher proportion of patients in unmet need agreed (*p* = 0.064).

**TABLE 4:** Comparison between met needs and unmet needs for ‘patient perception of quality’. (Ahmedabad, India. 2022).

	Met need (N = 141)	Unmet need (N = 91)	P value		Met need (N = 141)	Unmet need (N = 91)	P value
	Percent	Percent			Percent	Percent	
This hospital has all the medicines needed by you				You are able to get all the necessary medicines easily			
Disagree	6.4	5.5	0.357	Disagree	5.7	3.3	0.153
Neutral	2.1	0.0		Neutral	0.0	2.2	
Agree	91.5	94.5		Agree	94.3	94.5	
The doctors gave you advice about ways to avoid illness and stay healthy				The doctor gave you complete information about your illness			
Disagree	1.4	0.0	0.432	Disagree	1.4	0.0	0.242
Neutral	2.8	4.4		Neutral	0.0	1.1	
Agree	95.7	95.6		Agree	98.6	98.9	
The doctor gave you complete information about your treatment				Hospital workers talk politely			
Disagree	2.1	0.0	0.175	Disagree	14.2	11.0	0.726
Neutral	0.0	1.1		Neutral	4.3	5.5	
Agree	97.9	98.9		Agree	81.6	83.5	
Hospital workers are helpful to you				You are given enough time to tell the doctor everything			
Disagree	11.3	8.8	0.815	Disagree	2.1	1.1	0.524
Neutral	3.5	3.3		Neutral	2.1	4.4	
Agree	85.1	87.9		Agree	95.7	94.5	
Doctors listen carefully to what you have to say				The doctor checks patients properly			
Disagree	3.5	0.0	0.191	Disagree	1.4	0.0	0.375
Neutral	5.0	5.5		Neutral	0.7	0.0	
Agree	91.5	94.5		Agree	97.9	100.0	
The doctor is always ready to answer your questions				The doctor gave you adequate time			
Disagree	5.7	0.0	0.064	Disagree	3.5	1.1	0.259
Neutral	2.8	2.2		Neutral	3.5	1.1	
Agree	91.5	97.8		Agree	92.9	97.8	
The cleanliness of the hospital is adequate				The condition of the toilets are good			
Disagree	5.0	5.5	0.889	Disagree	9.9	9.9	0.672
Neutral	1.4	2.2		Neutral	2.8	1.1	
Agree	93.6	92.3		Agree	87.2	89.0	
Drinking water is easily available in the hospital				This hospital has all the requisite amenities			
Disagree	4.3	2.2	0.463	Disagree	2.8	0.0	0.174
Neutral	2.8	1.1		Neutral	1.4	3.3	
Agree	92.9	96.7		Agree	95.7	96.7	

**FIGURE 1:**
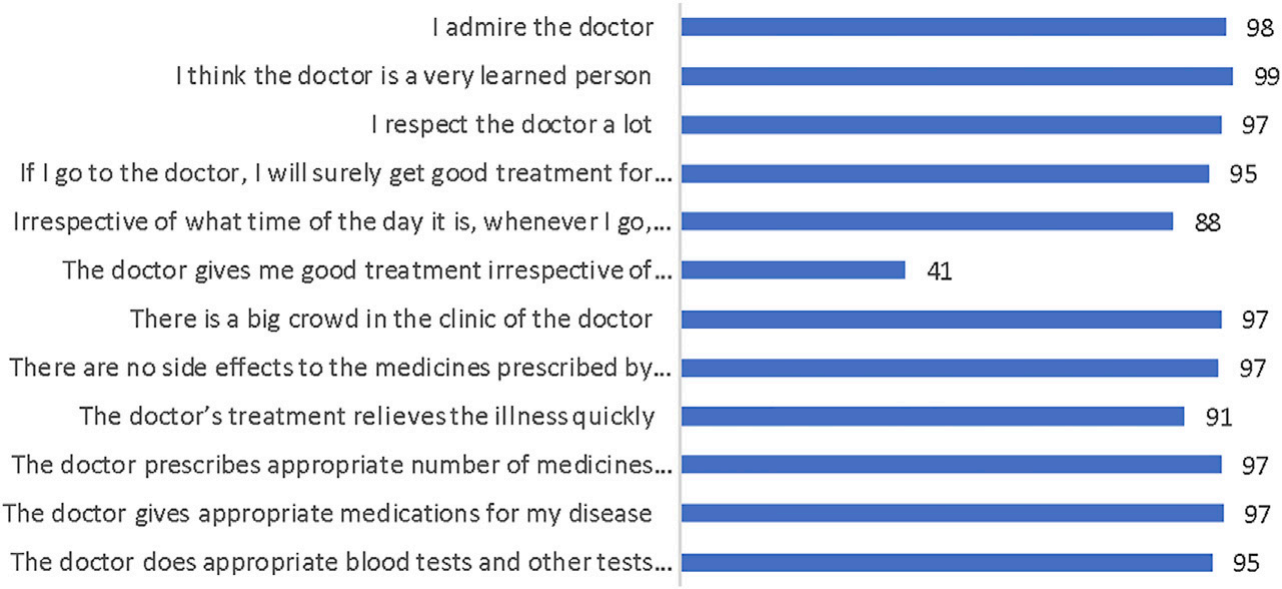
“Socio-culturally competent trust in physician scale for a developing country setting.” (Ahmedabad, India. 2022).

Figure 1 shows that the majority of participants (>90%) trusted doctors concerning investigations and prescription of medicines, and felt quick relief with the prescribed treatment. 97% indicated that the clinic was crowded, while only 41% agreed that the doctor treated them well irrespective of their paying capacity. More than 80% agreed that timings were flexible and the doctor can treat all illnesses while greater than 95% had faith in the skills of the doctor.

**FIGURE 2:**
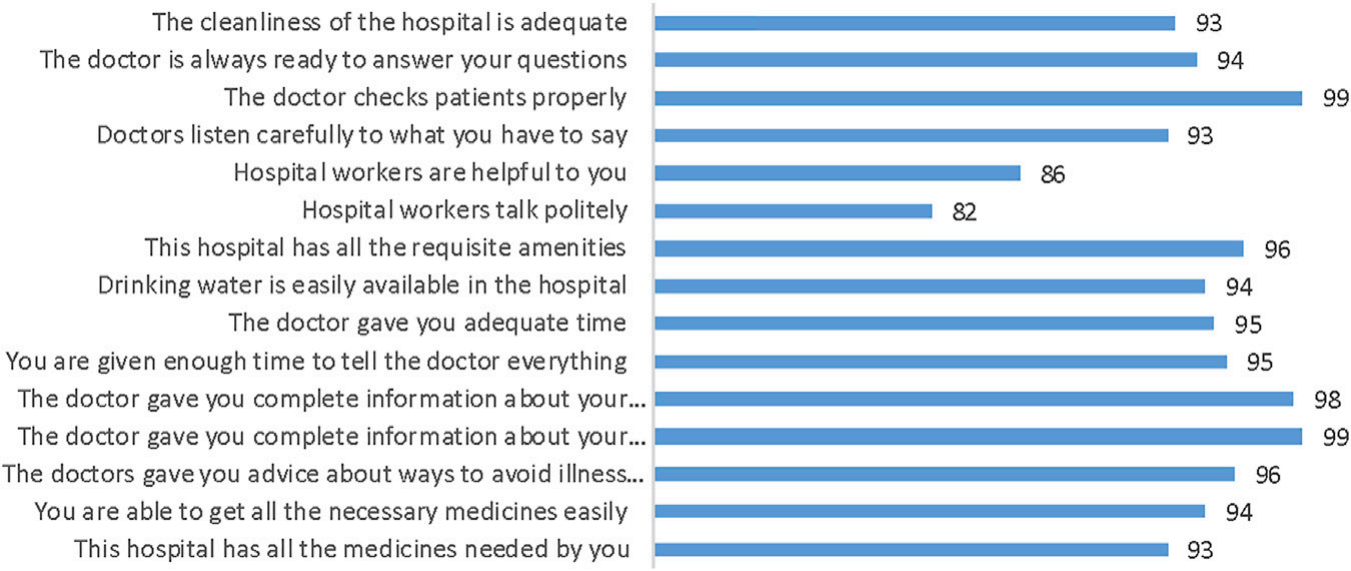
“Patient perceptions of quality” (Ahmedabad, India. 2022).

Figure 2 shows that greater than 90% of the participants were satisfied with the information/time provided by the doctor and the availability of the medicines/basic amenities. 82% of the participants indicated that hospital workers were polite and 86% indicated they were helpful.

Qualitative data from the in-depth interviews and focus group discussion validated the results from quantitative data. In both methods, participants agreed that there was high level of trust for the providers but felt that at times they were not given the depth of attention they expected. There was also a perception that multiple visits were required for relief of their symptoms which had financial implications. A male participant shared his experience and said, “During first visit, they told me my haemoglobin is low so I took 1-month course and went to the hospital. They told that you need to again take 1-month medicine course for haemoglobin. My haemoglobin increased to 12.5% so I asked for appointment for operation. They replied that they will give date after 15 days but they didn’t give me date and my problem increased.” A female participant said, “One doctor told with the help of medicine, you will recover. But it did not work so I went to another hospital and they operated immediately which caused great deal of stress and disruption of routine in the family.”

## Discussion

Our study showed the overall level of trust and perception of quality was high and patients held the doctors and hospital facilities in high regard. Met and unmet needs groups were similar in regards to trust and perception of quality. Less proportion of participants agreed that doctors treated them well irrespective of paying capacity. This indicates that finances affect trust and quality of care. Similar observations were made in the qualitative study using same domains as in quantitative data.

Trust in physicians is the unwritten covenant between patient and their physicians of mutual respect. A study in South India found that patients valued competence of the physician; behavior and assurance of treatment [15]. A study showed that trust in physicians was influenced by behavior on the physician and spending more time with the patient [16]. Giving patients time to explain the reason for visit, taking time to answer, enquiring about the family dynamics, involving them in decision-making and providing with medical information were determinants of trust in the physician and satisfaction [17].

Studies on user perceptions in LMIC have shown that patients were able to evaluate structural, process, and outcome measures of quality [18–21]. There is an evidence that doctors with better communication and interpersonal skills can detect problems, prevent medical crises and expensive interventions, while providing better support to their patients. This leads to better outcomes and satisfaction, lower costs, greater understanding of health issues, and adherence to the treatment process [22, 23]. We used 2 validated instruments, the Socio-culturally Competent Trust in Physician Scale and The Patient Perceptions of Quality in which majority of the participants (>90%) trusted doctors with respect to investigations, prescription of medicines and felt quick relief with the treatment. 97% indicated that the clinic was crowded, however, only 40% agreed that the doctor treated them well irrespective of their paying capacity.

Our previous work showed that financial constraints were the predominant reason for patients not availing of surgical care under UHC. Surveys carried out by the Government of India showed that the knowledge of UHC is patchy [24] and efforts have been made to increase education of patient rights and by automation [25]. However, we believe that there is widespread knowledge of UHC in Ahmedabad by the fact that almost 100% of patients obtained free surgical consultation and associated investigations. We assume that the financial constraints are a potential loss of wages during hospitalization. The majority of patients in our study were daily wagers and unlikely to have alternative means of support if they take time off for hospitalization.

It has been well documented that women in India face extensive gender discrimination in access to healthcare. Researchers calculated the number of “missing” patients by looking at the difference between the actual number of women who visited the hospital and those that should have come, based on the 2011 census. Almost two-thirds of these visits were made by male patients vs. 37% visits by female patients [26]. Another study found that average health care expenditures were lower among women than men [27]. Gender disparity was even visible in new-borns in rural Uttar Pradesh, India. Perception of illness was significantly lower in households with female versus male new-borns [28]. An observational study examined gender disparities in seeking healthcare and in-home management of diarrhea, acute respiratory infections, and fever among 530 children in a rural community of West Bengal, India, found that girls were less likely to get oral rehydration solutions during diarrhea [29].

Government schemes seeks to bridge the gender gap in the use of healthcare services by addressing a key constraint in using health services, i.e., the cost of healthcare. The underrepresentation, low coverage rates, and substantially lower benefits make women disadvantaged which are compounded with the financial burden of healthcare.

In summary, using mixed methodology, we showed that trust deficit could be mitigated by improved doctor-patient communication, in particular, by the emphasis that all patients would be treated equally irrespective of their paying capacity. The persistence of gender disparities calls for interventions at a societal level. Gender discrimination in access to healthcare could be removed by deploying female community health care workers. We have initiated a program of training surgical community workers that would sensitize gender issues. Financial toxicity needs to be removed at the governmental level by providing assistance to cover lost wages during hospitalization.

## Supplementary Material

Supplementary Appendix S1 Instruments used for quantitative study. (Ahmedabad, India. 2022).

Supplementary Appendix S2 Questions asked in the qualitative study. (Ahmedabad, India. 2022).

## Abbreviations

FGD, focused group discussion; LMIC, low and middle-income countries; UHC, universal health coverage.
