# Supplementary material for: Trust Deficit in Surgical Systems in an Urban Slum in India Under Universal Health Coverage: A Mixed Method Study
Source: Int J Public Health. 2022 Jul 14;67:1604924. doi: 10.3389/ijph.2022.1604924 (PMC9334907; doi:10.3389/ijph.2022.1604924)
Supplement: Supplementary file 1 [file DataSheet1.PDF]

APPENDIX 1
INSTRUMENT 1: SOCIO-CULTURALLY COMPETENT TRUST IN PHYSICIAN SCALE

**Item Scale**	**1. Strongly disagree**<br>**2. Disagree**<br>**3. Neutral**<br>**4. Agree**<br>**5. Strongly agree**
The doctor does appropriate blood tests and other tests to diagnose my disease	
The doctor gives appropriate medications for my disease	
The doctor prescribes appropriate number of medicines based on the nature of the illness	
The doctor prescribed more expensive medicines for serious illnesses	
The doctor’s treatment relieves the illness quickly	
The illness gets relieved with just one visit, there is no need for repeat visits	
There are no side effects to the medicines prescribed by the doctor	
Friends, relatives and neighbours speak well about the treatment provided by the doctor	
Friends, relatives and neighbours recommend me to go to the doctor	
I get the confidence that all my illness will get alright when I go to the doctor	
There is a big crowd in the clinic of the doctor	
If I go to the doctor, I will surely get good treatment for my illness	
The doctor gives me good treatment irrespective of whether I have money to pay	
The main intention of the doctor is to treat my illness and not anything else	
Irrespective of what time of the day it is, whenever I go, I can get good treatment from the doctor	
Whatever illness I have, I will go only to this doctor	
Even if I go to another doctor, I will take the treatment only if this doctor approves it	
I will bring my family members only to this doctor	
I will recommend only this doctor to all those who ask me	
I respect the doctor a lot	
I think the doctor is a very learned person	
I admire the doctor	

INSTRUMENT 2: PATIENT PERCEPTIONS OF QUALITY

**Item Scale**	**1. Strongly disagree**<br>**2. Disagree**<br>**3. Neutral**<br>**4. Agree**<br>**5. Strongly agree**
This hospital has all the medicines needed by you	
You are able to get all the necessary medicines easily	
The doctors gave you advice about ways to avoid illness and stay healthy	
The doctor gave you complete information about your illness	
The doctor gave you complete information about your treatment	
Hospital workers talk politely	
Hospital workers are helpful to you	
You are given enough time to tell the doctor everything	
Doctors listen carefully to what you have to say	
The doctor checks patients properly	
The doctor is always ready to answer your questions	
The doctor gave you adequate time	
The cleanliness of the hospital is adequate	
The condition of the toilets are good	
Drinking water is easily available in the hospital	
This hospital has all the requisite amenities	
