# Supplementary material for: Trust Deficit in Surgical Systems in an Urban Slum in India Under Universal Health Coverage: A Mixed Method Study
Source: Int J Public Health. 2022 Jul 14;67:1604924. doi: 10.3389/ijph.2022.1604924 (PMC9334907; doi:10.3389/ijph.2022.1604924)
Supplement: Supplementary file 2 [file DataSheet2.docx]

**Appendix 2: Patient Centred Questionnaire**

1. Which health issue led you to consult with the physician?
2. How long have you been aware of your health issue?
3. Have you previously consulted a physician about this health issue previously?

If Yes, what was the outcome?

1. Did the physician appear familiar with any of your previous health issues leading up to the consultation?
2. Did the physician take time to gather information relating to your social situation?
3. Did you fully understand what the physician when they explained your health issues and treatment options?
4. What did the physician tell you about your treatment options to address your health issues?
5. Were you given an opportunity to ask questions about your treatment options?
6. Were your personal circumstances (e.g. work and family commitments) taken consideration in the putting together of the treatment?
7. Did the physician refer you to another allied healthcare professional as part of your consultation?

If so, did the allied healthcare professional appear to be familiar with your case?

1. Were you given a point of contact (e.g. case manager or key person) to whom you could address any questions after the consultation?

If so, did you contact them and was the response satisfactory?

1. Did you decide to proceed with the treatment suggested by the physician?
2. If yes, what were the deciding factors in deciding to proceed with treatment?

If no, what were the deciding factors in deciding not to proceed with treatment
